# Integrated CDK4/6 inhibitor resistance and primary-metastatic discordance in hormone receptor-positive/HER2-negative metastatic breast cancer: a translational framework focused on invasive ductal carcinoma with gastric metastasis

**DOI:** 10.3389/fonc.2026.1918231

**Published:** 2026-10-06

**Authors:** Minyi Cheng, Dongqing Wang, Yingyi Lin, Cangui Wu, Ruobing Hong, Juanjuan Zhang, Jieqing Li

**Affiliations:** 1 Department of Breast Cancer, Cancer Center, Guangdong Provincial People’s Hospital (Guangdong Academy of Medical Sciences), Southern Medical University, Guangzhou, China; 2 School of Medicine, South China University of Technology, Guangzhou, China; 3 Department of Breast Oncology, Jieyang People’s Hospital, Jieyang, China; 4 Guangdong Provincial People’s Hospital Clinical Medical College, Southern Medical University, Guangzhou, China; 5 Department of Rehabilitation Medicine, Guangdong Provincial People’s Hospital (Guangdong Academy of Medical Sciences), Southern Medical University, Guangzhou, China

**Keywords:** CDK4/6 inhibitor, endocrine resistance, gastric metastasis, HER2-negative breast cancer, HR-positive breast cancer, invasive ductal carcinoma, metastatic discordance

## Abstract

**Background:**

CDK4/6 inhibitors have reshaped treatment for hormone receptor-positive (HR+)/HER2-negative metastatic breast cancer, but resistance frequently emerges and may interact with metastatic phenotypic evolution. We developed an integrated resistance-discordance framework and explored its clinical interpretability in invasive ductal carcinoma (IDC) with gastric metastasis.

**Methods:**

Public MSK-INK4 (cBioPortal study ID: breast_ink4_msk_2021) and MBCproject (brca_mbcproject_wagle_2017) cohorts were curated for HR+/HER2-negative disease. A five-module CDK4/6 inhibitor resistance score (0-5) was constructed from predefined mutation and copy-number events. In MBCproject RNA-seq samples, single-sample gene-set enrichment analysis quantified proliferation and estrogen-response activity. Discordance burden was defined as z(proliferation score) minus z(ER score), using primary tumors as the reference. Three institutional IDC gastric-metastasis cases were projected onto the framework for interpretability but not inferential testing.

**Results:**

Seventy HR+/HER2-negative MBCproject samples and 1116 deduplicated MSK-INK4 metastatic samples were analyzed. MBCproject metastases had higher resistance scores than primary tumors (P = 0.001), whereas metastatic scores were similar between MBCproject and MSK-INK4 (P = 0.202). Metastases showed higher proliferation activity (P = 0.014) and a trend toward lower ER activity (P = 0.071). The high-resistance/high-discordance quadrant was enriched for PI3K-axis events (OR = 16.52; P = 0.034), chromatin-remodeling loss-of-function events (OR = 12.47; P = 0.001), and cell-cycle/RB-axis events (OR = 9.03; P = 0.001). Among the three IDC gastric-metastasis cases, two available gastric NGS profiles mapped to predefined resistance modules, and the case with increased gastric Ki-67 showed the strongest IHC-adapted discordance and projected toward the high-resistance/high-discordance phenotype.

**Conclusion:**

Coupling resistance-associated genomic events with metastatic phenotype discordance may provide a clinically interpretable lens for HR+/HER2-negative metastatic breast cancer and for rare IDC gastric-metastasis presentations.

## Introduction

Endocrine therapy combined with cyclin-dependent kinase 4/6 (CDK4/6) inhibition is a major standard for HR+/HER2-negative advanced breast cancer, supported by large randomized trials of palbociclib, ribociclib, and abemaciclib (1–3). Despite durable benefit in many patients, acquired resistance remains common. Resistance is biologically heterogeneous: loss of intact RB signaling, cyclin E-CDK2 activation, endocrine escape, PI3K-AKT-mTOR activation, receptor tyrosine kinase/MAPK bypass, and chromatin-regulatory alterations can each reduce dependence on the canonical ER-cyclin D-CDK4/6 axis (4–8).

Metastatic breast cancer also evolves phenotypically. Receptor conversion between primary and metastatic lesions is well recognized, and transcriptomic studies have shown that metastatic samples may diverge from the primary tumor along proliferation, endocrine-response, immune, and lineage-state axes (9–12). Genomic profiling of paired biopsies before and after CDK4/6 inhibitor progression has further shown that resistance-associated alterations can accumulate in metastatic lesions and may coexist with transcriptomic shifts in proliferation and endocrine-response programs (13, 14). However, genomic resistance events and primary-metastatic phenotype discordance are often evaluated separately. An integrated framework that jointly summarizes resistance-associated genomic burden and metastatic phenotype deviation may provide a more interpretable view of metastatic adaptation in HR+/HER2-negative disease.

Gastric metastasis from breast cancer is rare and diagnostically challenging. Lobular carcinoma is classically overrepresented, but IDC can also metastasize to the stomach and may mimic primary gastric malignancy by clinical, endoscopic, or histologic appearance (15–17). In this setting, limited gastric biopsy material often needs to be interpreted using a combination of clinical history, immunohistochemistry, and targeted genomic profiling. Therefore, IDC gastric metastasis provides a clinically relevant scenario in which to examine whether a resistance-discordance framework can organize sparse but meaningful metastatic biopsy data.

We therefore constructed a five-module CDK4/6 inhibitor resistance score from public HR+/HER2-negative metastatic breast cancer cohorts and integrated it with RNA-based metastatic discordance metrics in the MBCproject cohort. We further evaluated whether high-resistance/high-discordance tumors were enriched for specific resistance modules and projected three institutional IDC gastric-metastasis cases onto the resulting framework as a clinical interpretability exercise, without using them for statistical inference.

## Materials and methods

### Study design and cohorts

This translational study combined public cohort analysis with an institutional interpretability case set. Public data were obtained from cBioPortal (18, 19). The MSK-INK4 cohort (breast_ink4_msk_2021) was used primarily to assess metastatic resistance-module frequency and score distribution, whereas the MBCproject cohort (brca_mbcproject_wagle_2017) (20) provided genomic data, lesion annotation, and RNA-seq data for resistance-discordance integration. Samples were restricted to HR+/HER2-negative breast cancer. In MSK-INK4, a maximum gene-panel priority strategy was used for deduplication. In MBCproject, samples were deduplicated at the patient level by preferentially retaining one primary and one metastatic sample; if multiple metastatic samples were available, a gastric metastasis was prioritized, otherwise the earliest metastatic biopsy was retained. Samples with ambiguous lesion-class information were counted in the initial HR+/HER2-negative screening set but were excluded from analyses requiring primary-versus-metastasis classification, including primary-metastasis comparisons, primary-reference z-score estimation, RNA-based discordance analysis, and resistance-discordance quadrant assignment.

### Resistance score construction

A sample-level CDK4/6 inhibitor resistance score was built from five predefined modules: cell-cycle/CDK axis, ER/endocrine resistance axis, PI3K-AKT-mTOR axis, RTK/MAPK bypass axis, and chromatin remodeling/epigenetic axis (**Table 1**). The gene and event list was defined *a priori* based on published mechanisms of endocrine and CDK4/6 inhibitor resistance and was not modified after inspecting the datasets. Loss-of-function mutations were defined as nonsense mutation, frameshift deletion, frameshift insertion, splice-site mutation, translation-start-site mutation, or nonstop mutation. Hotspot/oncogenic mutation status was assigned from original hotspot annotation and predefined canonical hotspots. Amplification was defined as discrete CNA = 2, and deep deletion as discrete CNA = -2. Non-hotspot missense mutations, synonymous variants, noncoding variants, low-level copy-number gains, and shallow deletions were excluded from the primary score. Each module contributed 1 point if at least one predefined event was present; multiple events within the same module were not counted repeatedly. Genes without eligible events in a given cohort contributed zero points and were not removed from the predefined scoring framework. The total score ranged from 0 to 5 (4–8). To evaluate sensitivity to this module-level rule, two alternative exploratory scores were also calculated: an event-count score, in which all eligible predefined events were counted individually, and a module-size-normalized event burden, in which the number of eligible events within each module was divided by the number of predefined genes in that module before summing across modules.

**Table 1:** Predefined gene modules and event rules for the CDK4/6 inhibitor resistance score (0-5).

Resistance module	Gene	Event definition	Data source
Cell-cycle/CDK axis	RB1	LoF mutation or deep deletion	Mutation + CNA
Cell-cycle/CDK axis	CCNE1	Amplification	CNA
Cell-cycle/CDK axis	CDKN2A	LoF mutation or deep deletion	Mutation + CNA
Cell-cycle/CDK axis	CDKN2B	LoF mutation or deep deletion	Mutation + CNA
Cell-cycle/CDK axis	CDK6	Amplification	CNA
Cell-cycle/CDK axis	CCND1	Amplification	CNA
ER/endocrine resistance axis	ESR1	Hotspot/oncogenic mutation	Mutation
ER/endocrine resistance axis	FGFR1	Amplification	CNA
PI3K-AKT-mTOR axis	PIK3CA	Hotspot/oncogenic mutation	Mutation
PI3K-AKT-mTOR axis	PTEN	LoF mutation or deep deletion	Mutation + CNA
PI3K-AKT-mTOR axis	AKT1	Hotspot/oncogenic mutation, especially AKT1 E17K	Mutation
PI3K-AKT-mTOR axis	RICTOR	Amplification	CNA
RTK/MAPK bypass axis	ERBB2	Amplification or hotspot/oncogenic mutation	Mutation + CNA
RTK/MAPK bypass axis	ERBB3	Amplification or hotspot/oncogenic mutation	Mutation + CNA
RTK/MAPK bypass axis	ERBB4	Amplification or hotspot/oncogenic mutation	Mutation + CNA
RTK/MAPK bypass axis	KRAS	Activating/hotspot mutation	Mutation
RTK/MAPK bypass axis	NRAS	Activating/hotspot mutation	Mutation
RTK/MAPK bypass axis	BRAF	Activating/hotspot mutation, especially BRAF V600	Mutation
Chromatin remodeling/epigenetic axis	KMT2C/MLL3	LoF mutation or deep deletion	Mutation + CNA
Chromatin remodeling/epigenetic axis	ARID1A	LoF mutation or deep deletion	Mutation + CNA
Chromatin remodeling/epigenetic axis	CHD4	LoF mutation or deep deletion	Mutation + CNA

LoF mutation was defined as nonsense mutation, frameshift deletion, frameshift insertion, splice-site mutation, translation-start-site mutation, or nonstop mutation. Amplification was defined as discrete CNA = 2, and deep deletion as discrete CNA = -2. Each module was scored once if at least one predefined event was present.

### RNA-based phenotype discordance

MBCproject RNA expression matrices were processed at the gene-symbol level. Duplicate gene symbols were averaged, genes with all missing values were removed, and non-log-distributed expression values were transformed as log2(x + 1). Single-sample gene-set enrichment analysis was performed with GSVA/ssGSEA (21). Proliferation activity was estimated using Hallmark E2F targets and G2M checkpoint gene sets, and ER pathway activity was estimated using MSigDB Hallmark estrogen response gene sets (22). Using primary tumors as the reference group, z(proliferation score) and z(ER score) were computed for each sample. Discordance burden was defined as z(proliferation score) - z(ER score), where higher values represent a shift toward higher proliferation and lower ER activity.

### Resistance-discordance integration

Samples with complete resistance score and RNA-derived discordance metrics were included in the integrated MBCproject analysis. Spearman rank correlation was used to evaluate associations among resistance score, z(proliferation score), z(ER score), and discordance burden. Median resistance score and median discordance burden were used to classify samples into four quadrants: low resistance/low discordance (Q1), low resistance/high discordance (Q2), high resistance/low discordance (Q3), and high resistance/high discordance (Q4). Module enrichment was tested using Fisher exact tests comparing Q4 with Q1-Q3 combined.

### Institutional IDC gastric-metastasis interpretability set

Three patients with primary breast IDC/non-special type and gastric involvement or gastric metastasis were retrospectively identified and summarized in **Supplementary Table 1**. Inclusion required evidence supporting gastric involvement/metastasis, available CDK4/6 inhibitor treatment history with clinical benefit or resistance/progression label, and paired primary-gastric immunohistochemistry sufficient for cross-site comparison. Formalin-fixed, paraffin-embedded tissue was tested on the BenchMark XT platform with ER SP1/Roche, PR 1E2/Roche, HER2/C-erbB2 4B5/Roche, and Ki-67 30-9/Roche antibodies. Two gastric NGS reports were available from clinical targeted panels and were used only to map events to predefined resistance modules. For the three-case projection, Delta Prolif was defined as gastric-metastasis Ki-67 minus primary Ki-67; Delta ER was defined as gastric-metastasis ER score minus primary ER score, where ER score = ER-positive percentage x staining intensity/3. z(Delta Prolif), z(Delta ER), and IHC-adapted discordance burden were calculated only for case projection. These cases were not included in public-cohort statistical inference.

### Statistical analysis

Two-group continuous comparisons used two-sided Wilcoxon rank-sum (Mann-Whitney U) tests; multi-group comparisons used Kruskal-Wallis tests with pairwise Wilcoxon comparisons where indicated in the figure legends. Associations among resistance score, z(proliferation score), z(ER score), and discordance burden were evaluated with Spearman rank correlation. For correlation analyses, false discovery rate (FDR)-adjusted P values were calculated using the Benjamini-Hochberg method within each analysis panel. Module enrichment in Q4 versus Q1-Q3 combined was tested with two-sided Fisher exact tests; odds ratios (ORs) and 95% confidence intervals (CIs) were reported where estimable. No multiple-comparison adjustment was applied unless stated in the figure legend. All tests were two-sided, and P < 0.05 was considered statistically significant. Analyses were performed in R (version 4.4.1) using GSVA for ssGSEA, ComplexHeatmap/maftools or equivalent packages for OncoPrint visualization, and standard statistical and ggplot2-based visualization packages.

## Results

### Public cohort assembly established a resistance-discordance analytic framework

The workflow is summarized in **Figure 1**. After HR+/HER2-negative filtering and deduplication, the MBCproject cohort included 70 samples. Of these, 34 primary tumors and 32 metastases had unambiguous lesion-class annotation and were used for primary-versus-metastasis comparisons and primary-reference RNA z-score estimation. Four samples had ambiguous lesion-class information and were excluded from analyses requiring primary-versus-metastasis classification. The integrated resistance-discordance analysis was further restricted to samples with complete genomic and RNA metrics and unambiguous lesion classification. The MSK-INK4 cohort included 1365 HR+/HER2-negative metastatic breast cancer samples before deduplication and 1116 metastatic samples after deduplication. Genomic events were curated into five resistance modules, and MBCproject RNA data were used to derive proliferation, ER-activity, and discordance-burden metrics.

**Figure 1:**
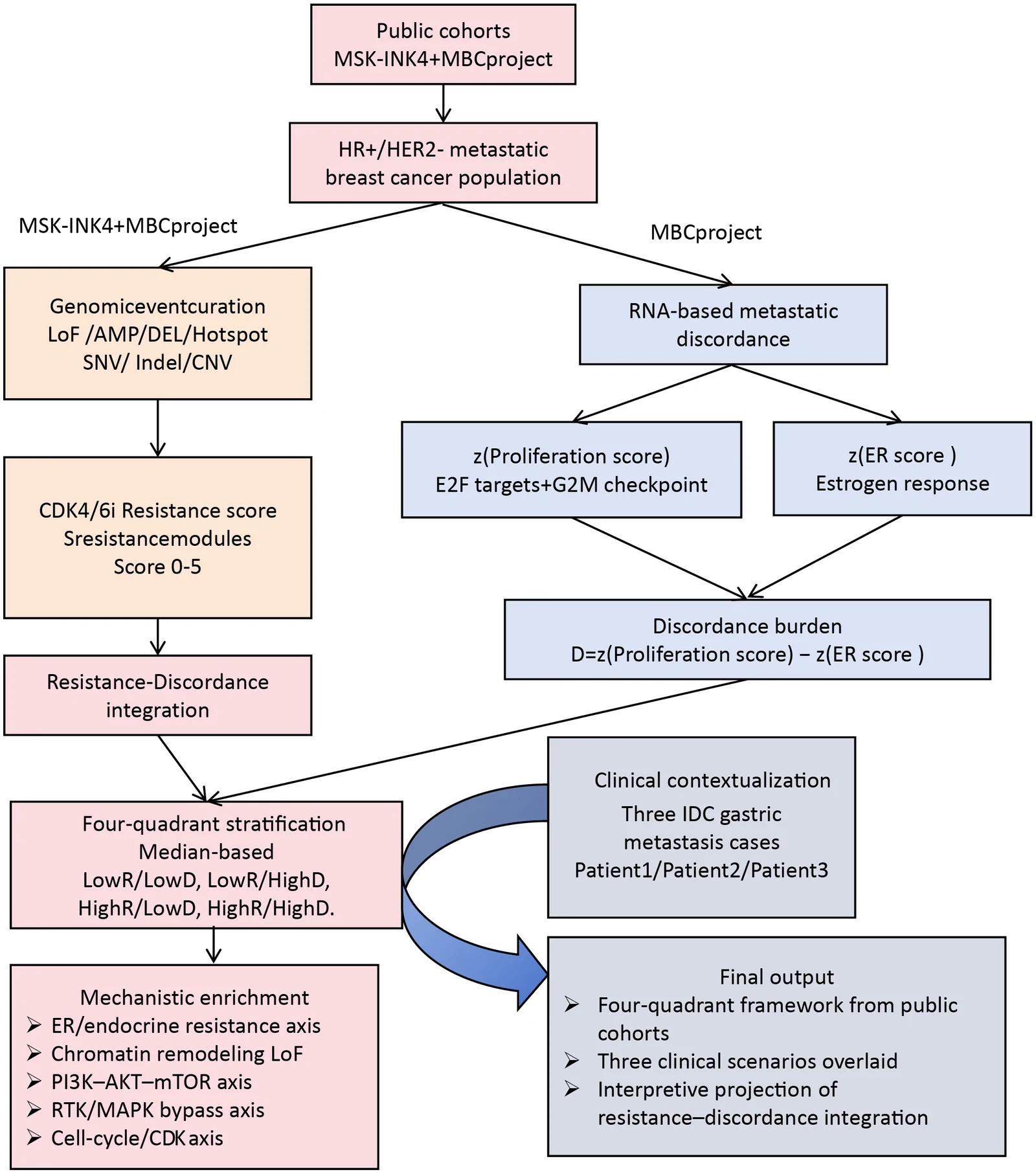
Study workflow for public cohort curation, CDK4/6 inhibitor resistance scoring, RNA-based metastatic discordance assessment, four-quadrant resistance-discordance integration, and clinical projection of IDC gastric-metastasis cases. HR, hormone receptor; IDC, invasive ductal carcinoma; ssGSEA, single-sample gene-set enrichment analysis; CNA, copy-number alteration; NGS, next-generation sequencing.

### Metastatic tumors accumulated more resistance-module events and higher resistance scores than primary tumors

The MBCproject OncoPrint showed recurrent alterations across the five predefined resistance modules, with metastatic samples displaying broader accumulation of cell-cycle/CDK, PI3K-AKT-mTOR, RTK/MAPK, and chromatin-remodeling/epigenetic events than primary tumors (**Figure 2A**). In the larger MSK-INK4 metastatic cohort, similar module classes were observed, with frequent CCND1 amplification, PIK3CA mutation, ESR1 mutation, FGFR1 amplification, and recurrent chromatin-remodeling gene events (**Figure 2B**). These findings supported the use of a module-level resistance score rather than reliance on a single gene alteration. In MBCproject, metastatic samples had significantly higher resistance scores than primary tumors (P = 0.001; **Figure 3**). The score distribution of MBCproject metastases was not significantly different from that of MSK-INK4 metastases (P = 0.202), suggesting that the smaller MBCproject metastatic subset captured a resistance-burden range broadly consistent with the larger MSK-INK4 metastatic cohort.

**Figure 2:**
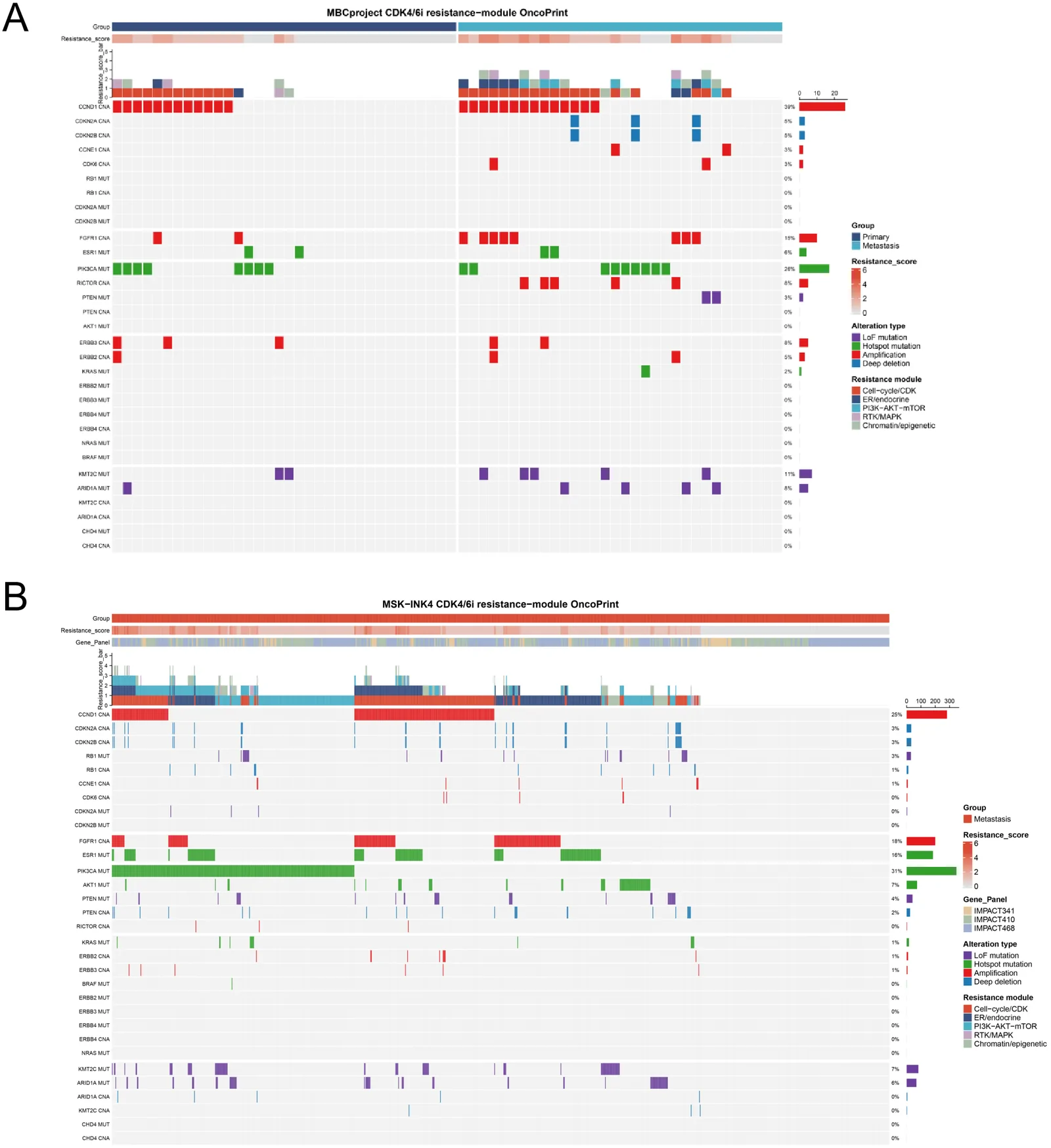
CDK4/6 inhibitor resistance-module OncoPrints. **(A)** MBCproject primary (n = 34) and metastatic (n = 32) HR+/HER2-negative samples. **(B)** Deduplicated MSK-INK4 HR+/HER2-negative metastatic samples (n = 1116). Predefined mutation and copy-number events from cBioPortal were grouped by alteration type and resistance module; only events included in the five-module resistance score are shown. LoF, loss-of-function. Genes without observed scoring events were retained in the OncoPrints because the gene/event list was predefined *a priori*; these rows contributed zero points to the resistance score.

**Figure 3:**
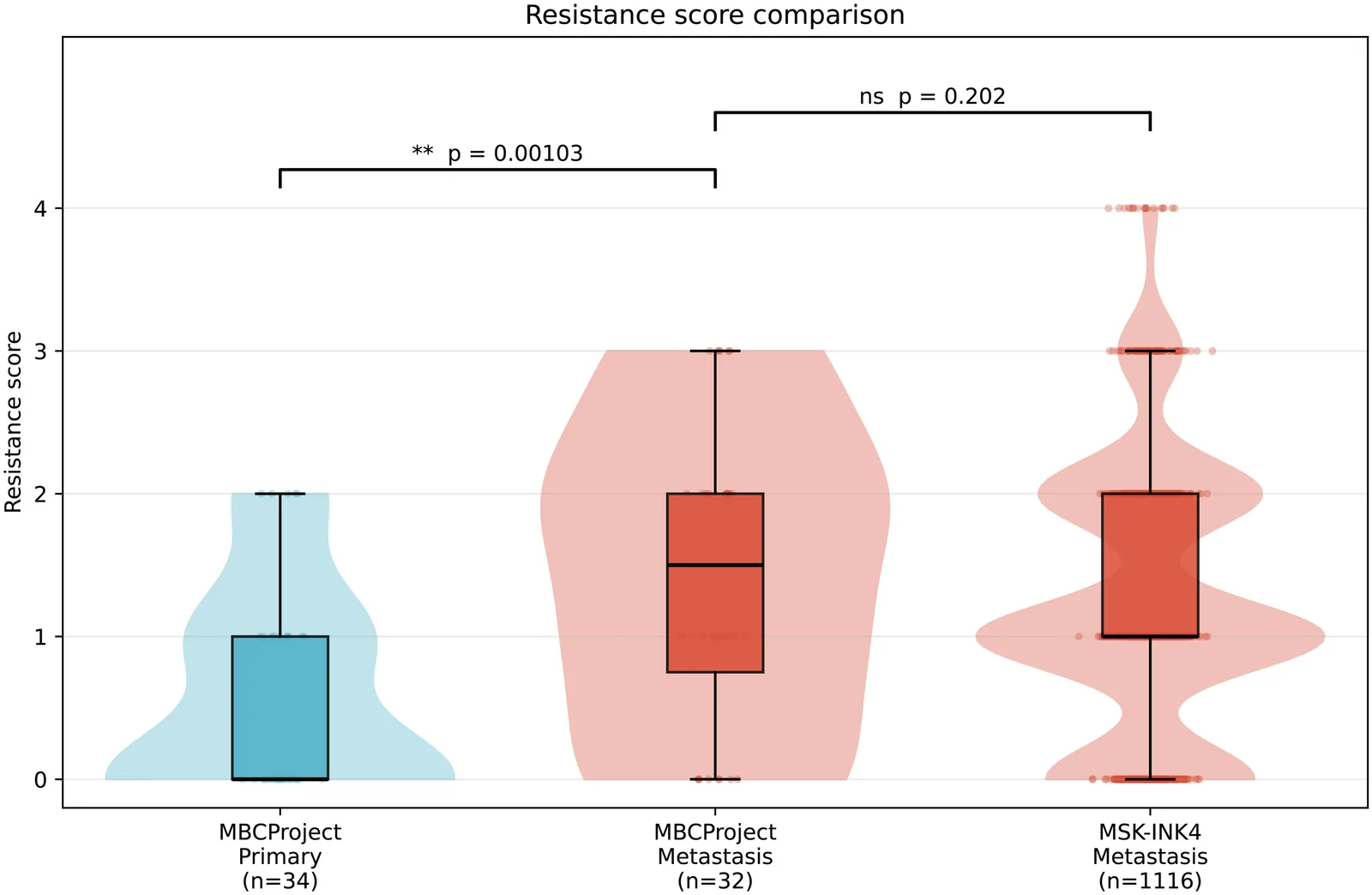
Resistance score comparison across MBCproject primary tumors (n = 34), MBCproject metastases (n = 32), and MSK-INK4 metastases (n = 1116). Box plots show the module-level CDK4/6 inhibitor resistance score (range, 0-5). Two-sided Wilcoxon rank-sum tests were used for pairwise comparisons. MBCproject metastases showed higher resistance scores than primary tumors (P = 0.001), while MBCproject and MSK-INK4 metastases were not significantly different (P = 0.202). **P<0.01.

### Metastases shifted toward higher proliferation and lower er activity relative to primary tumors

ssGSEA-based pathway scoring showed higher proliferation activity in metastases than in primary tumors (P = 0.014; **Figure 4A**). ER activity tended to be lower in metastases, although this comparison did not reach conventional statistical significance (P = 0.071; **Figure 4A**). When z(proliferation score) and z(ER score) were plotted jointly, a subset of metastatic samples occupied a high-proliferation/low-ER-activity region, supporting the discordance-burden definition (**Figure 4B**). A ranked heatmap further showed that high discordance burden was generally driven by positive proliferation deviation with reduced or relatively lower ER activity (**Supplementary Figure 1**).

**Figure 4:**
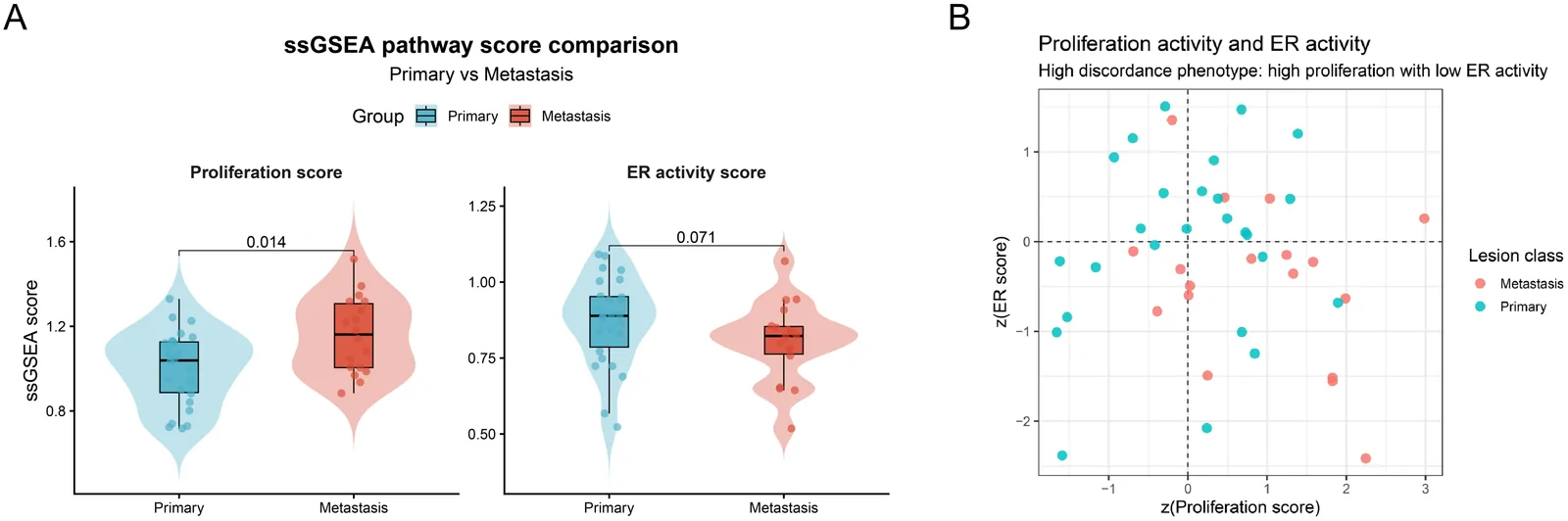
RNA-based phenotype discordance in MBCproject. **(A)** ssGSEA pathway scores for proliferation (Hallmark E2F targets and G2M checkpoint) and ER activity (Hallmark estrogen response early) in primary (n = 34) versus metastatic (n = 32) samples. Two-sided Wilcoxon rank-sum tests were used. **(B)** Two-dimensional scatter plot of z(proliferation score) versus z(ER score), with primary tumors used as the reference for z-score calculation. Discordance burden was defined as z(proliferation score) minus z(ER score). ER, estrogen receptor.

### Integrated resistance-discordance analysis identified a Q4 subset enriched for PI3K, cell-cycle/RB, and chromatin-remodeling events

In integrated MBCproject samples with complete genomic and RNA metrics (n = 44), Spearman correlation analysis showed that the resistance score was positively correlated with z(proliferation score) (rho = 0.484, P < 0.001, FDR = 0.003) and discordance burden (rho = 0.383, P = 0.010, FDR = 0.015), whereas its correlation with z(ER score) was weak and nonsignificant (rho = -0.110, P = 0.477, FDR = 0.477; **Supplementary Table 2**). Sensitivity analyses using event-count and module-size-normalized scoring approaches showed similar positive relationships with discordance burden, supporting the robustness of the module-level scoring rule (**Supplementary Table 2**). We also benchmarked the resistance score against previously reported CDK4/6i resistance-related mTORC1 and MYC target signatures, but no significant correlations were observed (**Supplementary Table 2**).

Median-based stratification separated samples into four biologically interpretable resistance-discordance groups (**Figure 5A**; **Supplementary Table 3**). Q4 represented the high-resistance/high-discordance subset. Compared with all other quadrants combined, Q4 was enriched for PI3K-axis events (OR = 16.52; P = 0.034), chromatin-remodeling loss-of-function events (OR = 12.47; P = 0.001), and cell-cycle/RB-axis events (OR = 9.03; P = 0.001; **Figure 5B**). RTK/MAPK and ER-module enrichment showed similar directional trends but did not reach statistical significance (RTK/MAPK: OR = 5.32, P = 0.11; ER module: OR = 4.3, P = 0.16). The all-quadrant enrichment bubble plot further supported the preferential distribution of PI3K, cell-cycle/RB, and chromatin-remodeling events in Q4 (**Supplementary Figure 2**).

**Figure 5:**
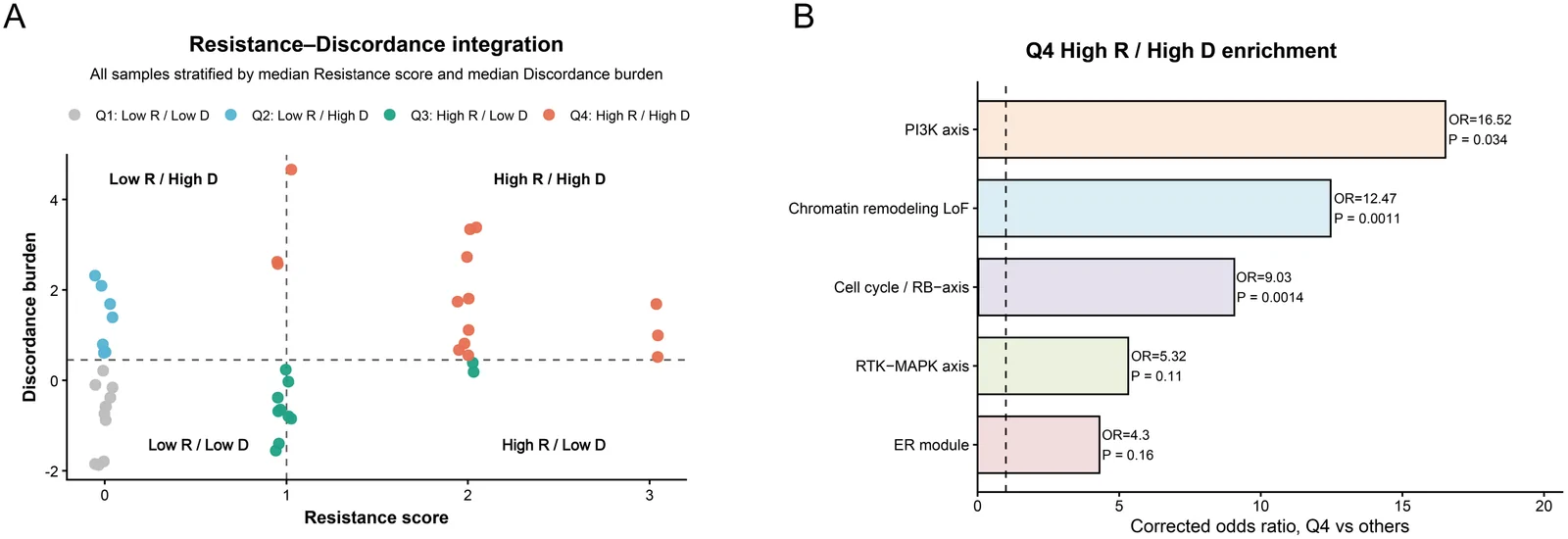
Resistance-discordance integration in MBCproject samples with complete genomic and RNA metrics. **(A)** Median resistance score and median discordance burden were used to define four quadrants: Q1, low resistance/low discordance; Q2, low resistance/high discordance; Q3, high resistance/low discordance; Q4, high resistance/high discordance. **(B)** Enrichment of predefined resistance modules in Q4 versus Q1-Q3 combined, tested by two-sided Fisher exact test; bubble size reflects OR and color reflects P value.

### IDC gastric-metastasis cases demonstrated clinical projectability of the resistance-discordance framework

Three institutional IDC cases with gastric involvement or gastric metastasis were projected onto the resistance-discordance framework (**Figure 6**; **Supplementary Table 1**). Patient 1 was clinically resistant to ribociclib plus letrozole but lacked matched molecular coordinates; IHC-adapted projection placed the case near the cohort median discordance boundary. Patient 2 was clinically resistant to ribociclib plus exemestane and had a chromatin-remodeling/epigenetic resistance-module event, but the IHC-adapted discordance metric was low because gastric Ki-67 decreased relative to the primary tumor. Patient 3, treated with ribociclib-based endocrine therapy and labeled resistant, mapped to the high-resistance/high-discordance quadrant; NGS showed RICTOR and ARID1A module events, and the IHC-adapted metrics showed increased proliferation deviation with reduced ER deviation (**Supplementary Figure 3**). These cases illustrate how clinical, IHC, and NGS data can be organized within the resistance-discordance coordinate system while preserving their noninferential role. Together, these cases provide a descriptive clinical projection of the resistance-discordance framework using available IHC and NGS information.

**Figure 6:**
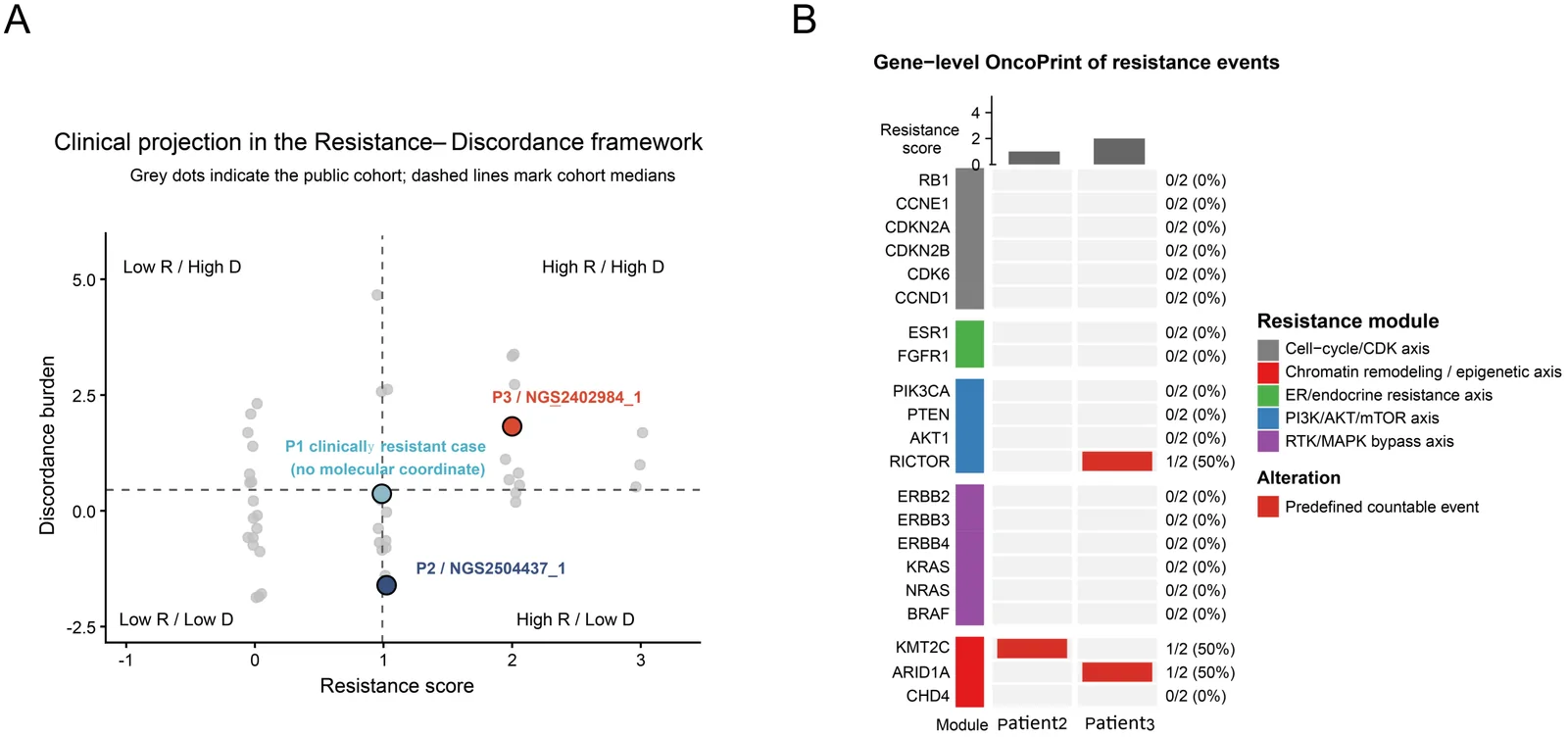
Clinical projection of three institutional IDC gastric-metastasis cases into the resistance-discordance framework. Cases were mapped using IHC-adapted discordance metrics and available NGS module events; they were not included in public-cohort statistical inference. Gene-level OncoPrint shows resistance-module events in the two NGS-profiled gastric metastases. IDC, invasive ductal carcinoma; IHC, immunohistochemistry; NGS, next-generation sequencing.

## Discussion

This study proposes a clinically oriented resistance-discordance framework for HR+/HER2-negative metastatic breast cancer. By combining a curated five-module CDK4/6 inhibitor resistance score with RNA-based primary-metastatic phenotype deviation, we found that metastatic samples carried higher resistance scores than primary tumors and that a high-resistance/high-discordance subset was enriched for PI3K, cell-cycle/RB, and chromatin-remodeling events. Projection of three IDC gastric-metastasis cases showed how the same framework may help organize sparse but clinically meaningful IHC and NGS data. This study provides a reproducible, clinically oriented interpretability framework that integrates predefined CDK4/6 inhibitor resistance-related genomic modules with RNA-based phenotype discordance.

A module-level score is biologically plausible because CDK4/6 inhibitor resistance is convergent rather than monogenic. RB loss can directly impair CDK4/6 inhibitor dependence, whereas CCNE1 amplification and CDK2 activation may bypass G1 restriction. ESR1 mutations, FGFR1 amplification, PI3K pathway activation, RTK/MAPK signaling, and chromatin remodeling alterations can each remodel endocrine dependence or proliferative state (4–8, 13, 14, 23).Counting modules rather than individual events reduces over-weighting of highly altered loci within the same pathway and produces a simple score that can be applied across targeted-panel datasets.

The RNA component adds information that genomic scoring alone cannot capture (9–14). A tumor can harbor resistance-associated events but retain strong ER transcriptional activity, or conversely show substantial loss of ER-response activity without many predefined genomic events. Discordance burden operationalizes this distinction as a high-proliferation/low-ER-activity shift relative to primary tumors. This phenotype is clinically relevant because endocrine sensitivity depends not only on receptor positivity by IHC but also on functional ER pathway dependence and cell-cycle output. RNA-derived discordance and IHC-adapted discordance should therefore be viewed as complementary rather than interchangeable readouts: RNA-based scores reflect pathway-level transcriptional activity, whereas ER and Ki-67 IHC provide clinically accessible approximations of ER expression and proliferative status. The small institutional case set precluded formal testing of whether IHC-based proliferation/ER patterns reproduce the RNA-derived discordance pattern.

We also benchmarked the genomic resistance score against previously reported CDK4/6 inhibitor resistance-related pathway signatures (24, 25), including mTORC1 signaling and MYC target programs. No significant correlations were observed, suggesting that the score captures a complementary genomic-event layer rather than simply reflecting these transcriptomic pathway activities.

The enrichment of PI3K, cell-cycle/RB, and chromatin-remodeling events in Q4 is especially noteworthy. PI3K pathway alterations can support endocrine escape and are therapeutically actionable in selected HR+/HER2-negative metastatic breast cancer contexts (23).Cell-cycle/RB-axis events directly affect the pharmacologic target network of CDK4/6 inhibitors. Chromatin-remodeling events may influence lineage state, transcriptional plasticity, and endocrine response (5, 8). The Q4 phenotype therefore may represent a state in which genomic bypass mechanisms and transcriptomic endocrine attenuation coexist.

Gastric metastasis provides a useful clinical stress test for the framework. Breast cancer metastasis to the stomach is uncommon, can present long after the primary tumor, and may be difficult to distinguish from primary gastric carcinoma without careful IHC and clinical correlation (15–17). Although lobular carcinoma is often emphasized, the three cases here involved IDC/non-special type. Patient 3, which mapped to the high-resistance/high-discordance quadrant and carried PI3K/chromatin events, illustrates how a rare metastatic presentation can be interpreted through the same axes used for public cohort analysis.

This study has several limitations. First, the analysis was retrospective and correlative, and the MBCproject RNA subset was modest with limited matched primary-metastatic pairs; therefore, discordance burden was defined using a primary-reference rather than strictly patient-paired strategy. Second, an independent external cohort with matched genomic and transcriptomic profiles was not included, and the framework requires validation in larger cohorts with treatment exposure and clinical outcome data. Third, public datasets had incomplete CDK4/6 inhibitor exposure and response annotations, so the resistance score should be interpreted as a resistance-associated molecular burden rather than a validated predictor of treatment failure. In addition, a larger matched IHC-genomic cohort was not available, so the relationship between IHC-adapted discordance and RNA-derived discordance could not be formally tested. Finally, the institutional IDC gastric-metastasis cases were used only for interpretability and were not intended for statistical inference or clinical validation.

## Conclusion

An integrated CDK4/6 inhibitor resistance-discordance framework identified a high-resistance/high-discordance subset of HR+/HER2-negative metastatic breast cancer enriched for PI3K, cell-cycle/RB, and chromatin-remodeling events. The framework also provided a practical structure for interpreting IDC gastric-metastasis cases with limited IHC and NGS data. Prospective, paired-biopsy, and functional studies are needed before clinical decision-making use.

## Data Availability

The original contributions presented in the study are included in the article/**Supplementary Material**. Further inquiries can be directed to the corresponding authors.
