# Supplementary material for: Integrated CDK4/6 inhibitor resistance and primary-metastatic discordance in hormone receptor-positive/HER2-negative metastatic breast cancer: a translational framework focused on invasive ductal carcinoma with gastric metastasis
Source: Front Oncol. 2026 Oct 6;16:1918231. doi: 10.3389/fonc.2026.1918231 (PMC13680221; doi:10.3389/fonc.2026.1918231)
Supplement: Supplementary file 1 [file Table1.docx]

**Supplementary Table S1. Clinically annotated IDC cases with gastric involvement used for interpretability (not for inference).**

| **Variable** | **Patient 1** | **Patient 2** | **Patient 3** |
| --- | --- | --- | --- |
| Age, years | 53 | 40 | 57 |
| Sex | Female | Female | Female |
| Case role label | Resistance anchor | Resistance anchor | Discordance anchor |
| Histology | Invasive ductal carcinoma | Invasive ductal carcinoma | Invasive ductal carcinoma |
| IHC surrogate subtype | Luminal B1-like | Luminal B1-like | Luminal B-like |
| Evidence of gastric involvement/metastasis | Gastroscopic biopsy of gastric body lesions showed poorly differentiated metastatic carcinoma; ER 3+, 95%; PR 1+, <1%; HER2 2+ without amplification; Ki-67 approximately 30%, supporting breast origin. | Gastroscopic biopsy showed a gastric body tumor; CK+++, GATA3+++, and weak mammaglobin positivity supported gastric metastasis from breast cancer. | Gastric antrum biopsy showed poorly differentiated carcinoma; CK7++, CK20-, CDX2-, ER 95%, 3+, supporting gastric metastasis from breast cancer. |
| Primary ER | ER 90%, 2+ | ER 90%, 2+ | ER 100%, 3+ |
| Primary PR | Negative | PR 90%, 2+ | PR 10%, 2+ |
| Primary HER2 | Negative | Negative | Negative |
| Primary Ki-67 | 30% | 50% | 30% |
| Gastric metastasis ER | ER 95%, 3+ | ER 90%, 3+ | ER 95%, 3+ |
| Gastric metastasis PR | Negative | PR 55%, 1-2+ | PR 5%, 1+ |
| Gastric metastasis HER2 | Negative | Negative | Negative |
| Gastric metastasis Ki-67 | 30% | 10% | 35% |
| Delta Ki-67, percentage points | 0 | -40 | 5 |
| CDK4/6i regimen and clinical outcome label | Ribociclib + letrozole; resistant | Ribociclib + exemestane; resistant | Ribociclib-based endocrine therapy; resistant |
| Gastric tissue NGS report ID | NA | NGS2504437 | NGS2402984 |
| Key NGS events mapped to resistance modules | NA | Chromatin remodeling/epigenetic axis | PI3K/AKT/mTOR axis; chromatin remodeling/epigenetic axis |
