# Supplementary material for: Integrated CDK4/6 inhibitor resistance and primary-metastatic discordance in hormone receptor-positive/HER2-negative metastatic breast cancer: a translational framework focused on invasive ductal carcinoma with gastric metastasis
Source: Front Oncol. 2026 Oct 6;16:1918231. doi: 10.3389/fonc.2026.1918231 (PMC13680221; doi:10.3389/fonc.2026.1918231)
Supplement: Supplementary file 2 [file Table2.docx]

**Supplementary Table S2. Spearman correlation and sensitivity analyses supporting the resistance-discordance framework.**

| **Analysis** | **Comparison** | **n** | **Spearman rho** | **P value** | **FDR** | **Direction** |
| --- | --- | --- | --- | --- | --- | --- |
| ***A. Primary Spearman correlations with the original resistance score*** | | | | | | |
| A | Resistance score vs z(Proliferation score) | 44 | 0.484 | <0.001 | 0.003 | Positive |
| A | Resistance score vs z(ER score) | 44 | -0.110 | 0.477 | 0.477 | Negative |
| A | Resistance score vs Discordance burden | 44 | 0.383 | 0.010 | 0.015 | Positive |
| ***B. Sensitivity analyses using alternative scoring approaches*** | | | | | | |
| B | Event-count score vs Discordance burden | 44 | 0.428 | 0.004 | 0.009 | Positive |
| B | Event-count score vs z(Proliferation score) | 44 | 0.490 | <0.001 | 0.002 | Positive |
| B | Event-count score vs z(ER score) | 44 | -0.150 | 0.331 | 0.331 | Negative |
| B | Module-size-normalized event burden vs Discordance burden | 44 | 0.372 | 0.013 | 0.018 | Positive |
| B | Module-size-normalized event burden vs z(Proliferation score) | 44 | 0.368 | 0.014 | 0.018 | Positive |
| B | Module-size-normalized event burden vs z(ER score) | 44 | -0.190 | 0.216 | 0.243 | Negative |
| B | Module-size-normalized module score vs Discordance burden | 44 | 0.371 | 0.013 | 0.018 | Positive |
| B | Resistance score vs event-count score | 44 | 0.842 | <0.001 | <0.001 | Positive |
| B | Resistance score vs module-size-normalized event burden | 44 | 0.794 | <0.001 | <0.001 | Positive |
| ***C. Benchmark correlations with mTORC1 and MYC signatures*** | | | | | | |
| C | Resistance score vs z(HALLMARK_MTORC1_SIGNALING) | 44 | -0.157 | 0.309 | 0.716 | Negative |
| C | Resistance score vs z(HALLMARK_MYC_TARGETS_V1) | 44 | -0.008 | 0.961 | 0.970 | Negative |
| C | Resistance score vs z(HALLMARK_MYC_TARGETS_V2) | 44 | -0.117 | 0.448 | 0.716 | Negative |
| C | Discordance burden vs z(HALLMARK_MTORC1_SIGNALING) | 44 | 0.132 | 0.391 | 0.716 | Positive |
| C | Discordance burden vs z(HALLMARK_MYC_TARGETS_V1) | 44 | 0.195 | 0.205 | 0.716 | Positive |
| C | Discordance burden vs z(HALLMARK_MYC_TARGETS_V2) | 44 | 0.190 | 0.217 | 0.716 | Positive |
| C | z(Proliferation score) vs z(HALLMARK_MYC_TARGETS_V1) | 44 | 0.063 | 0.683 | 0.910 | Positive |
| C | z(Proliferation score) vs z(HALLMARK_MTORC1_SIGNALING) | 44 | -0.006 | 0.970 | 0.970 | Negative |

Note: n indicates the number of integrated MBCproject samples included in each correlation analysis. Spearman rho represents Spearman's rank correlation coefficient. P values are nominal two-sided values; FDR values are Benjamini-Hochberg adjusted values within the corresponding analysis panel. Direction indicates the sign of the correlation coefficient. ER, estrogen receptor; FDR, false discovery rate.
