# Supplementary material for: Integrated CDK4/6 inhibitor resistance and primary-metastatic discordance in hormone receptor-positive/HER2-negative metastatic breast cancer: a translational framework focused on invasive ductal carcinoma with gastric metastasis
Source: Front Oncol. 2026 Oct 6;16:1918231. doi: 10.3389/fonc.2026.1918231 (PMC13680221; doi:10.3389/fonc.2026.1918231)
Supplement: Supplementary file 3 [file Table3.docx]

**Supplementary Table S3. Sample-level resistance-discordance metrics and quadrant assignments in integrated MBCproject samples.**

| **Sample ID** | **Resistance score** | **Event count** | **Module-size normalized event burden** | **Module-size normalized module score** | **Discordance burden** | **z(Prolif)** | **z(ER)** | **Quadrant** |
| --- | --- | --- | --- | --- | --- | --- | --- | --- |
| MBC-MBCProject_zatdFvhp-Tumor-SM-GQAAR | 3 | 3 | 1.000 | 1.000 | 0.516 | 0.024 | -0.492 | Q4: High R / High D |
| MBC-MBCProject_RKf1frsr-Tumor-SM-CGLJI | 3 | 3 | 0.750 | 0.750 | 1.687 | 1.331 | -0.357 | Q4: High R / High D |
| MBC-MBCProject_DxibF0hM-Tumor-SM-CGLWP | 3 | 5 | 1.167 | 0.833 | 0.993 | 0.802 | -0.191 | Q4: High R / High D |
| MBC-MBCProject_Y7iLIOU0-Tumor-SM-GQBY8 | 2 | 2 | 0.333 | 0.333 | 0.814 | 1.290 | 0.476 | Q4: High R / High D |
| MBC-MBCProject_nwIeUoSD-Tumor-SM-GQC2D | 2 | 3 | 0.750 | 0.500 | 0.672 | 0.747 | 0.076 | Q4: High R / High D |
| MBC-MBCProject_nkU3hDhB-Tumor-SM-GQC49 | 2 | 2 | 0.500 | 0.500 | 1.112 | 0.944 | -0.169 | Q4: High R / High D |
| MBC-MBCProject_3MUnT7Ty-Tumor-SM-CGLLE | 2 | 4 | 0.750 | 0.333 | 0.186 | 1.388 | 1.202 | Q3: High R / Low D |
| MBC-MBCProject_xBfJfri9-Tumor-SM-CGLAP | 2 | 2 | 0.583 | 0.583 | 3.340 | 1.824 | -1.515 | Q4: High R / High D |
| MBC-MBCProject_wAiri7fp-Tumor-SM-AZ5DH | 2 | 2 | 0.833 | 0.833 | 0.388 | -0.388 | -0.776 | Q3: High R / Low D |
| MBC-MBCProject_QbiZiytx-Tumor-SM-CGMH7 | 2 | 2 | 0.500 | 0.500 | 3.381 | 1.826 | -1.555 | Q4: High R / High D |
| MBC-MBCProject_PzUBH7I3-Tumor-SM-CGLNW | 2 | 3 | 0.917 | 0.417 | 0.553 | 1.034 | 0.480 | Q4: High R / High D |
| MBC-MBCProject_KphMt4F9-Tumor-SM-DL4PB | 2 | 3 | 0.833 | 0.667 | 2.727 | 2.985 | 0.258 | Q4: High R / High D |
| MBC-MBCProject_57iLiJIl-Tumor-SM-CGLIV | 2 | 3 | 0.917 | 0.667 | 1.740 | 0.248 | -1.492 | Q4: High R / High D |
| MBC-MBCProject_4MF1FlFQ-Tumor-SM-CGM4M | 2 | 2 | 0.500 | 0.500 | 1.807 | 1.581 | -0.226 | Q4: High R / High D |
| MBC-MBCProject_wKuwTYhK-Tumor-SM-GQCRI | 1 | 1 | 0.167 | 0.167 | -0.684 | -1.525 | -0.841 | Q3: High R / Low D |
| MBC-MBCProject_QbiZiytx-Tumor-SM-CGM9N | 1 | 1 | 0.333 | 0.333 | -0.848 | -0.308 | 0.540 | Q3: High R / Low D |
| MBC-MBCProject_oqupfDu7-Tumor-SM-GQCOZ | 1 | 2 | 0.750 | 0.500 | -1.399 | -1.617 | -0.218 | Q3: High R / Low D |
| MBC-MBCProject_LDCbC8t9-Tumor-SM-GQCEB | 1 | 1 | 0.167 | 0.167 | -0.796 | 0.676 | 1.471 | Q3: High R / Low D |
| MBC-MBCProject_Jeu1F5cz-Tumor-SM-DL48Y | 1 | 1 | 0.167 | 0.167 | -0.646 | -1.657 | -1.011 | Q3: High R / Low D |
| MBC-MBCProject_glf3CWFa-Tumor-SM-GQ9N5 | 1 | 1 | 0.167 | 0.167 | -0.382 | 0.178 | 0.560 | Q3: High R / Low D |
| MBC-MBCProject_eXSVsxUm-Tumor-SM-GQC2Z | 1 | 1 | 0.167 | 0.167 | 0.236 | 0.494 | 0.258 | Q3: High R / Low D |
| MBC-MBCProject_6JhKimhv-Tumor-SM-GQCJC | 1 | 2 | 0.417 | 0.167 | 2.574 | 1.893 | -0.680 | Q4: High R / High D |
| MBC-MBCProject_ZeTySaU3-Tumor-SM-GQD3T | 1 | 3 | 0.500 | 0.167 | 2.622 | 1.990 | -0.632 | Q4: High R / High D |
| MBC-MBCProject_k4hlFwFg-Tumor-SM-GQA9I | 1 | 2 | 0.417 | 0.167 | -1.554 | -0.200 | 1.354 | Q3: High R / Low D |
| MBC-MBCProject_briXiduE-Tumor-SM-DL49L | 1 | 2 | 0.583 | 0.333 | 4.661 | 2.246 | -2.415 | Q4: High R / High D |
| MBC-MBCProject_8bfWIQu1-Tumor-SM-GQCD2 | 1 | 1 | 0.167 | 0.167 | -0.030 | 0.463 | 0.492 | Q3: High R / Low D |
| MBC-MBCProject_xlhkS5CG-Tumor-SM-GQ9ZP | 0 | 0 | 0.000 | 0.000 | -0.159 | -0.016 | 0.143 | Q1: Low R / Low D |
| MBC-MBCProject_qQSACYuv-Tumor-SM-GQBFZ | 0 | 0 | 0.000 | 0.000 | -0.101 | 0.380 | 0.480 | Q1: Low R / Low D |
| MBC-MBCProject_QNs8uGhM-Tumor-SM-GQCB6 | 0 | 0 | 0.000 | 0.000 | 2.091 | 0.844 | -1.247 | Q2: Low R / High D |
| MBC-MBCProject_qmu6TYto-Tumor-SM-GQA6Z | 0 | 0 | 0.000 | 0.000 | -0.741 | -0.595 | 0.146 | Q1: Low R / Low D |
| MBC-MBCProject_MYuoCmI7-Tumor-SM-DL4R7 | 0 | 0 | 0.000 | 0.000 | 0.794 | -1.588 | -2.382 | Q2: Low R / High D |
| MBC-MBCProject_KGs1fxH2-Tumor-SM-CGM1H | 0 | 0 | 0.000 | 0.000 | -0.576 | 0.329 | 0.905 | Q1: Low R / Low D |
| MBC-MBCProject_JGcLUJiA-Tumor-SM-GQBDH | 0 | 2 | 0.750 | 0.000 | 2.316 | 0.239 | -2.077 | Q2: Low R / High D |
| MBC-MBCProject_gjhMuoha-Tumor-SM-AXGGJ | 0 | 1 | 0.250 | 0.000 | -1.793 | -0.288 | 1.505 | Q1: Low R / Low D |
| MBC-MBCProject_gASyuQSl-Tumor-SM-GQ9KM | 0 | 1 | 0.500 | 0.000 | -1.871 | -0.932 | 0.939 | Q1: Low R / Low D |
| MBC-MBCProject_EkHAIECZ-Tumor-SM-GQ9OE | 0 | 0 | 0.000 | 0.000 | 1.688 | 0.680 | -1.008 | Q2: Low R / High D |
| MBC-MBCProject_DWTkcWCW-Tumor-SM-GQB7T | 0 | 0 | 0.000 | 0.000 | -0.880 | -1.165 | -0.285 | Q1: Low R / Low D |
| MBC-MBCProject_beTYUZij-Tumor-SM-GQBWY | 0 | 0 | 0.000 | 0.000 | -1.849 | -0.697 | 1.152 | Q1: Low R / Low D |
| MBC-MBCProject_B7f5hRfD-Tumor-SM-GQACN | 0 | 0 | 0.000 | 0.000 | 0.625 | 0.726 | 0.101 | Q2: Low R / High D |
| MBC-MBCProject_7JtRIPI2-Tumor-SM-CGMAW | 0 | 0 | 0.000 | 0.000 | -0.381 | -0.419 | -0.038 | Q1: Low R / Low D |
| MBC-MBCProject_zyt5TKFB-Tumor-SM-CGMBJ | 0 | 1 | 0.250 | 0.000 | 0.213 | -0.095 | -0.308 | Q1: Low R / Low D |
| MBC-MBCProject_XVHkUVTb-Tumor-SM-GQCLU | 0 | 2 | 0.417 | 0.000 | 0.606 | 0.008 | -0.598 | Q2: Low R / High D |
| MBC-MBCProject_Ali6SAHL-Tumor-SM-GQAH2 | 0 | 0 | 0.000 | 0.000 | 1.393 | 1.245 | -0.148 | Q2: Low R / High D |
| MBC-MBCProject_7of5i3hp-Tumor-SM-CGMAA | 0 | 0 | 0.000 | 0.000 | -0.582 | -0.690 | -0.108 | Q1: Low R / Low D |

Note: Values are shown for integrated MBCproject samples with complete genomic and RNA metrics (n = 44). Numeric continuous variables are rounded to three decimal places. Discordance burden was defined as z(proliferation score) - z(ER score). Quadrants were defined by median resistance score and median discordance burden. R, resistance; D, discordance; ER, estrogen receptor.
